# Patient and Public Involvement with Forced Migrants: An Empirical Exploration of Ethical Issues

**DOI:** 10.1007/s11673-025-10438-3

**Published:** 2025-08-12

**Authors:** Elin Inge, Nimo Elmi, Yasmin Omar, Georgina Warner, Ulrik Kihlbom

**Affiliations:** 1 CHAP, Department of Public Health and Caring Sciences, Uppsala University, Box 564, 751 22 Uppsala, Sweden; 2 Department of Learning, Informatics, Management and Ethics (LIME), Karolinska Institutet, Inst. LIME, Att: Ulrik Kihlbom, Karolinska Institutet, 171 77 Stockholm, Sweden

**Keywords:** Patient and public involvement, Forced migrants, Empirical ethics, Relational ethics, Qualitative, Thematic analysis

## Abstract

Patient and public involvement (PPI) with forced migrants can have positive impacts on health research, but empirical knowledge on the ethics of involving forced migrants is scarce. Unsolved ethical issues risk hindering meaningful involvement and jeopardize the research process, as well as harming the public contributors and causing moral distress. In this article, we aimed to identify ethical issues in PPI with forced migrants and present case examples, based on qualitative data and using thematic analysis, as well as analyse the issues using the ethical principles by Beauchamp and Childress as well as PPI-centred values. The ethical issues identified were “Treating forced migrant public contributors as a vulnerable group can inhibit autonomy”; “Non-inclusive communication strategies can contribute to injustice”; “Regulations around payment risk excluding the most vulnerable from involvement”; “Public contributors risk being excluded from partaking in decision-making,” and “If trust is not established, public contributors do not feel safe sharing honest input.” Further, we discussed the ethical issues using relational ethics, with a focus on how to conduct PPI with forced migrants in an ethical way.

## Background

Patient and public involvement (PPI) in research can lead to a richer understanding of the research topic and improve the research process and outcomes (Brett et al. 2014). However, this relies on public involvement being conducted in a meaningful, respectful, and inclusive way, especially when involving representatives from more seldomly involved populations such as forced migrants (Hickey et al. 2022). Here, we understand PPI as an active partnership between researchers and representatives of the group in focus, who together create and develop research. These representatives of a group, here termed *public contributors*, are not study participants but collaborators. They have lived experience relevant to the research in focus; in health research, patient partners are common but also representatives of societal groups. They represent the perspective of living in a situation, as opposed to studying it, and contribute with experiential knowledge to complement the academic knowledge. The public contributors can be active collaborators in all stages of the research cycle, from initial idea generation where public contributors can give input on research question and methods, through data collection and analysis, to dissemination and implementation of the findings where public contributors can develop material and reach non-academic audiences. In the literature, many other terms are used for similar approaches with a large variation between academic fields and geographical regions (Dengsø et al. 2023).

There are two main arguments for PPI. The first is the rights-based—or ethical—argument; the public has the right to be involved in research about them. Lucy Frith (2023) calls this a democratic argument and connects PPI to a global movement to widen democratic practices, outside of conventional forms of democracy. The second argument—the methodological argument—concerns the impact of PPI on research i.e., PPI makes research better. PPI evaluations on various aspects of research show impact such as increased relevance and research focus and improvements of research material and recruitment (Boote et al. 2010; Brett et al. 2014; Domecq et al. 2014; Røssvoll et al. 2022; Vat et al. 2019).

Many scholars have written about the problems in forming ethical relationships with forced migrants in research (Davidson, Hammarberg and Fischer, (Davidson et al., 2023); Gaywood et al. 2020; Hynie 2018; Mackenzie et al. 2007; Mollard et al. 2020; Vaughn et al. 2018), and it is reasonable to assume that these issues extend to PPI with forced migrants. The migration experience poses specific risks to one’s health (Hynie 2018) and forced migrants are more likely to experience inequities, such as racism and other societal systems of oppression (Hamed et al. 2022; King et al. 2022). This leads to epistemic injustice (Fricker 2007), where the experiences of forced migrants are not represented where research is conducted and knowledge is produced. This is true even when forced migration is the topic of research. Krause (2017) found that refugees are often deemed “invisible actors” in research, by which she argues that doing research *with* rather than *about* refugees could alleviate the asymmetry of power. Thus, involvement of forced migrants is crucial to make research about their life and health relevant and useful.

In a review of reviews and textbooks, Gradinger et al. (2015) mapped values associated with PPI and divided them into a normative value system and a substantive value system relating to *why* PPI should be done. The normative values focused on moral, ethical, and political values: empowerment, rights, change/action, accountability/transferability and a number of ethical values. The substantive values are related to PPI impact: effectiveness, quality/relevance, validity/reliability, representativeness/objectivity/generalizability, and evidence base. These value systems correspond well to the two arguments for PPI, with the normative value system representing the ethical argument, and the substantive value system representing the methodological argument. In addition to this, Gradinger et al. identified a process value system, with values relating to *how* PPI should be done: partnership/equality, respect/trust, openness/honesty, independence, and clarity (Gradinger et al. 2015).

Apart from this, health researchers must also adhere to research ethics principles. The principles of Beauchamp and Childress (2013) are widely used in health research: respect for autonomy, beneficence, non-maleficence, and justice. Additionally, both the Helsinki Declaration (World Medical Association 2013) and the Belmont Report (The National Commission for the Protection of Human Subjects of Biomedical and Behavioral Research, 1979) highlight the need for increased protection for “vulnerable groups” in which forced migrants are often included. Although these are not related to PPI per se, they guide researchers’ ethical work with research participants and influence the way they interact with non-researchers.

Ethical issues in PPI have been described in the literature through reporting on specific PPI activities and in reflective summaries. For example, Banks et al. (2013) outlined general ethical challenges in PPI. They found issues concerning partnerships and power in research relationships, blurred boundaries between researcher and researched, issues around meaningful representation of a community, data disseminations as well as issues around ownership of data and findings. In connection to traditional non-participatory research, they found issues around anonymity, confidentiality, and institutional ethical review processes. Several ethical frameworks have been developed to support the ethical conduct of PPI, for example Pandya-Wood et al.’s (2017) framework for ethical involvement in the design stage of health research. Another framework is written by Groot and Abma (2022), who map and discuss the “ethics work” of PPI, for example “emotion work” and “relationship work.”

Voices in the PPI field have argued that negative impact is under-reported (Russell et al. 2020). For PPI with forced migrants specifically, empirical knowledge on ethics is scarce. In a reflective article, James (2023) discusses the ethical tension in a participatory photovoice project with asylum seekers. Pincock and Bakunzi (2021) argue that although participatory methods have potential to contribute to equality, uncritically applied they instead risk masking top-down initiatives. Roura et al. (2021) draws upon literature and researcher experiences when showing the value of involving migrants in research while simultaneously pointing out potential barriers to meaningful involvement and the need for evaluating PPI initiatives. Unsolved ethical issues ultimately risk hindering meaningful involvement and jeopardize the research process, as well as harming the public contributors and causing moral distress (Jameton 1984) for participatory researchers (Pandya-Wood et al. 2017). Therefore, we saw a need to empirically investigate ethical issues in PPI with forced migrants. We aimed to identify ethical issues in PPI with forced migrants, present case examples, analyse the issues using ethical principles and values, discuss them and provide suggestions on how to conduct PPI in an ethical way.

## Method

The empirical basis for this article was the data from a public involvement evaluation project, in which involvement of forced migrants in health research was explored. The studies in this project are summarized briefly below and details can be found in the original publications. All studies had ethical approval from the Swedish Ethical Review Authority (reference numbers 2018/382; 2020/03911; 2020/03911; 2020/03126 and 2020/06693*).*

- Study 1: We tracked the experiences among refugee parents involved as public contributors in health research. Data consisted of longitudinal focus group interview data with refugee parent public contributors across a 3-year project on a trauma support programme for refugee children (Lampa et al. 2022).
- Study 2: We investigated enablers and barriers in research meetings with forced migrant public contributors in four health research projects. The data consisted of behavioural observations of group dynamics and decision-making processes in PPI research meetings, and survey data from the attending researchers and public contributors (Inge et al. 2023b).
- Study 3: We evaluated communication strategies used in a project on co-creating an online adaptation of a support group for youth with posttraumatic stress. The data consisted of behavioural observations of the project meetings, field notes and documented communication between team members (Inge et al. 2023a).

The ethical issues were identified from data and findings (Inge et al. 2023a, b; Lampa et al. 2022), as well as from a commentary on online PPI (Lampa et al. 2021), through secondary analysis. The first author’s field notes and ethical reflections from data collections and practical PPI work were also revisited. In a first step, thematic analysis according to Braun and Clarke (2021) was used. The first author read the original data, the published findings, field notes, and ethical reflections, repeatedly. Thereafter she created an initial list of themes related to ethical issues, in discussion with the author team. The case examples were extracted from the data and reformulated. In the second step, the themes and cases were reflected upon in relation to PPI values (Gradinger et al. 2015) and the ethical principles by Beauchamp and Childress (2013), together will the author team. This was done iteratively; themes were discussed and revised several times. In the discussion, we introduce relational ethics (Clandinin et al. 2018) as a way to understand the identified ethical issues.

This article was written in collaboration between academic researchers and public contributors with experiences of forced migration and of working with research. Initially, this work was conducted in online meetings, with open discussions on the aim of the study, the data, and initial reflections on ethical issues. The first author wrote a draft of the article, after which the authors met for a longer workshop to discuss the manuscript. Thereafter, a revised version was sent out together with a summary in Swedish, followed by two online meetings with further discussions. The public contributors were especially active in forming the discussion.

## Findings

We identified five themes of ethical issues in PPI with forced migrants. In this section, the five issues are presented (Table 1) and exemplified with real case examples.

**Table 1:** Ethical issues in PPI with forced migrants

Treating forced migrant public contributors as a vulnerable group can inhibit autonomy
Non-inclusive communication strategies can contribute to injustice
Regulations around payment risk excluding the most vulnerable from involvement
Public contributors risk being excluded from partaking in decision-making
If trust is not established, public contributors do not feel safe sharing honest input

### Treating Forced Migrant Public Contributors as a Vulnerable Group Can Inhibit Autonomy

Forced migrants can, according to ethical guidelines in research, be classified as a vulnerable group, which warrants increased considerations on the need for protection in major research ethics guidelines, such as the Helsinki Declaration (World Medical Association 2013) or the Belmont Report (The National Commission for the Protection of Human Subjects of Biomedical and Behavioral Research 1979). Although forced migrants in PPI are not research participants but colleagues of the researchers, their situational vulnerability might increase the risk for exploitation and harm. Researchers are aware of this and do consider the need for increased protection. However, in PPI, researchers need to balance this against both the public contributors’ autonomy as well as PPI values, such as partnership, empowerment, openness, and honesty (Gradinger et al. 2015). This leads to an ethical issue for researchers; when acting to protect forced migrant public contributors, researchers might in fact limit their possibilities to make their own decisions i.e., decrease the public contributors’ autonomy.

An example can be seen in this case, where a researcher acts to protect the public contributors involved in a project. The researcher was approached by an academic colleague about inviting the public contributors as research participants to another study, as the colleague was struggling to recruit participants with a forced migrant background. The researcher declined the request with two arguments; that the public contributors might not feel comfortable to say no due to their ongoing involvement and that they were not yet confident in their existing role and might struggle to separate this role from the role as research participants. Here, the researcher acted to protect the public contributors, having identified a risk for a situation where the public contributors would not be able to make an informed autonomous decision. Another course of action would have been to allow the public contributors to make the decision themselves, which would have been more in line with the principle of respect for public contributors’ autonomy. However, in this context, the researchers would need to be confident that the public contributors would be able to separate between their public contributor role and the research participant role to make an autonomous decision. Ultimately, this would rely on the researchers’ ability to communicate the options in an accessible way, which led to the choice to turn down the request i.e., to protect the public contributors. This can be seen as a paternalistic action, as the researchers made the decision for the public contributors rather than letting them decide themselves.

In another case example, researchers wanted to involve refugee parents in the steering committee for a research project on trauma support for refugee children. When recruiting parents to the project, the researchers hesitated around the ethical aspects of inviting refugees who had just arrived in Sweden. On one hand, newly arrived refugees would have ongoing experiences from life on arrival to Sweden, which would be valuable as the fast-changing migration policies in Sweden were affecting the life and health of asylum-seekers. In addition, PPI often contributes with value for the involved individual, for example with empowerment. On the other hand, the researchers were worried about the increased contextual vulnerability of newly arrived refugees and the appropriateness of asking them to take on yet another task, while navigating in their new context. In addition, those who had resided in Sweden for some time would have a better overview of the context for refugees in Sweden and how this unfolds over time. This would add value to the project, for example concerning in which phase of resettlement refugees should be offered trauma support. In this case, limited recruitment venues eventually led the researchers to involve refugee parents who had resided in Sweden one year or more. Once the refugee parents had started working in the project, the team discussed the pre-recruitment issues. The refugee contributors argued that it is not appropriate to involve refugees directly after arrival, as “life is chaos.” In such a chaotic situation, they said, a person cannot fully grasp the concept of involvement and do not know enough about their new society, for example laws, to be able to relate to it. Thereby, ethical involvement in research might not be possible. They suggested a year as a minimum time before becoming involved as a public contributor in research. In this case, protection was something that both researchers and public contributors agreed on as an important value.

### Non-Inclusive Communication Strategies Can Contribute to Injustice

A second ethical issue is non-inclusive communication strategies. This is not necessarily a matter of conflicting values or non-adherence to principles, rather lack of ethical involvement practices. Communicating in an inclusive and accessible way is an essential skill for the participatory researcher, to avoid that public contributors are excluded or miss important information on project context or process. Non-inclusive communication can lead to systems of epistemic injustice being reinforced, which is especially relevant for groups that experience inequities.

The non-inclusive communication strategies included the use of scientific jargon and acronyms. One example was researchers using technical terms such “light touch” or “non clinical population” when discussing mental health interventions. Apart from being difficult for the public contributors to understand, the terms were also challenging for the language interpreter to translate. If information is communicated in ways that are accessible for researchers—i.e., in a language they understand and using scientific jargon—but in ways that cause barriers for the public contributors—i.e., abbreviations, scientific terminology—issues around justice arise.

In addition, parts of the observed projects were conducted during the COVID-19 pandemic, which prompted online meetings. Here, we observed less social interaction, which was associated with less in-depth discussions and less input, and an increase in linguistic barriers. This can lead to limited insight to the project for public contributors, which raises issues around their autonomy; a prerequisite for involvement is that public contributors are fully informed and able to make autonomous decisions around becoming and staying involved. Ethical PPI requires clear and comprehensible communication, as stated in the PPI values accountability/transparency. It is also a prerequisite to obtain process values such as partnership/equality and clarity.

One additional layer, especially relevant for newly arrived forced migrants, is communicating through a language interpreter. This makes the researchers and contributors reliant on them and their skills to obtain process-oriented values such as partnership, independence, and clarity. Without proper interpretation, the public contributors risk missing out on receiving the same information as the rest of the team, thus lacking real possibilities for clarity, as well as not having their input well translated, thus being hindered to provide relevant input.

In a case example, we recorded an ethically concerning incident with an interpreter overtaking the discussion by expressing own ideas and actively attempting to affect the public contributors’ input. Due to the language barrier, the researchers did not fully grasp the situation until afterwards. This left the public contributors exposed to the interpreter’s communication, leading to ethical concerns around their actual possibilities to gain meaningful insight to the project discussions and contribute on their own terms. Eventually, post-meeting discussions with the public contributors led to another interpreter being employed. We acknowledge this is not always possible, which raised a concern that unethical conduct by interpreters might occur unnoticed. Ethical risks might be exacerbated when researchers are not used to working with interpreters, as this requires an adapted mode of communication such as pausing more often and planning for time for translation in a meeting.

### Regulations Around Payment Risk Excluding the Most Vulnerable From Involvement

The third ethical issue has a practical nature: it revolves around financial reimbursement for public contributors. The authors’ stance—which aligns with recommendations for PPI (NIHR 2022)—is that all public contributors should be financially reimbursed for their work, just as the researchers are. The main argument for this is justice: everyone’s time, effort, and knowledge should be valued. While financial reimbursement is not yet standard practice, public contributors were reimbursed in the studies observed for this article. There were, however, issues in reimbursing the most vulnerable public contributors, which caused moral distress (Jameton 1984) for the responsible researchers.

In the studies, public contributors were paid an hourly wage, according to a fixed local rate informed by the INVOLVE guidelines (NIHR 2022). However, a complication was that the forced migrant public contributors had different legal statuses of residence in Sweden. Public contributors with a permanent residence permit were eligible for payment according to the university’s regular system. Regulations to reimburse those with temporary residence permits, those who resided in Sweden undocumented, or those with social benefits was more difficult to navigate. This led to practical difficulties in reimbursing them for their time, in a way that was legal, benefitted them, and did not put them at risk for future taxation issues or issues with the Migration Agency. All public contributors also experienced administrative difficulties in accessing the information needed to actually receive their reimbursement, even those eligible for regular payment. Therefore, practical and legal issues in reimbursing all forced migrants equally, makes the principle of justice difficult to adhere to for researchers. The universities’ systems are not built for financial reimbursement of public contributors and functions as a barrier to involve those who are not Swedish speakers, highly educated, and integrated.

One solution would be to only involve forced migrants who are eligible for regular payment. However, this also raises issues in relation to the principle of justice, as this would mean the active exclusion of those who are—at a group level—more vulnerable. This goes against core PPI values such as representativeness, partnership/equality, and respect/trust. This contributed to moral distress (Jameton 1984) among the researchers in the projects.

### Public Contributors Risk Being Excluded From Partaking in Decision-Making

This fourth ethical issue is process-oriented and relates to how decisions are made. A key factor in PPI, especially to ensure that collaborations are meaningful and not tokenistic, is that decisions are taken together. Shared decision-making and the reallocation of power from the researchers to the public contributors, aligns with core PPI values, including partnership and equality, as well as the principle of justice. However, involvement can be done in different ways. In a team, all members, including researchers, have different skill sets; every team member is not necessarily expected to be involved in all decisions. The contributor’s role is often described as somewhat different and a common position is that they should be involved in most decision-making processes.

In our observations of PPI meetings, we noted when and how decisions were taken and if and how public contributors were involved in them. Surprisingly, decisions were rarely taken in the steering committee meetings, where the forced migrant public contributors were present. From the researcher notes, we learned that decisions were often taken in-between meetings by the academic researchers. This decision-making procedure had not been discussed or actively decided on by the team. In the interviews with refugee parents involved as public contributors, we saw that they were aware and accepting of this dynamic; they phrased it as “we share our experiences—researchers decide what is useful.” In the data there were also some good examples of shared decision-making, especially during smaller meetings in close collaborations; however, it was the occasions where public contributors were excluded from decision-making that stood out as an ethical issue.

There are several factors complicating how decision-making should be done. One factor is responsibility; there might be legal restrictions for the responsible researcher to share decision-making with the public contributors. Another factor is balancing which decisions should be shared and which should in fact be taken by researchers; there might, for example, be methodological decisions where experience-based knowledge is not so relevant. In addition, it is likely that researchers are so used to working in a specific way, that they simply do not reflect over decisions being taken without consulting the public contributors. Finally, we need to ask ourselves: Do researchers actually want to share decision-making and to what extent? Here, we argue that a researcher’s commitment to working with PPI holds them accountable to share decision-making in a way that is mutually agreed upon and discuss this with the public contributors in a transparent manner.

### If Trust is Not Established, Public Contributors Do Not Feel Safe Sharing Honest Input

The final ethical issue concerns the development of trust, especially if and how public contributors develop trust in the researchers and the research process. In our definition of trust, we draw upon the Stanford Encyclopedia of Philosophy (2020): “Trust is an attitude we have towards people whom we hope will be trustworthy, where trustworthiness is a property not an attitude.” Further, the definition states that trust requires that we can be vulnerable to others, rely on others to be competent to do what we wish to trust them to do, and rely on them to be willing to do it. In addition, we want to acknowledge that trust is a multi-dimensional concept (Freitag and Traunmüller 2009; Gandolfo 2022), meaning that trust is present at different levels and there are multiple trust relationships existing at the same time; between public contributors and researchers, public contributors and other public contributors, public contributors and the institution, etc. In this study, we also drew upon the concepts “generalized trust”, i.e. an abstract trust that is placed in strangers, and “particularized trust,” an intimate trust in those in the immediate network, such as family, friends, and co-workers (Freitag and Traunmüller 2009). Establishing trust is essential to attain PPI values such as empowerment and quality/relevance but also process-oriented PPI values including equality, respect, honesty, and independence (Gradinger et al. 2015). In the observed projects, we saw several examples of the importance of trust—and the risks when trust was not established. While this ethical issue is not a matter of conflicting values, it identifies a poor condition for working in an ethical way.

In one example, forced migrants involved as public contributors shared that their journey to trust the researchers had taken a long time; they stated that trust had been established after two to three years of collaboration. However, even after three years, one refugee public contributor stated they were “not yet fully safe, deep down” and that the collaboration should have been longer and more intense (Lampa et al. 2022). A concern if trust is not established, is that PPI collaborations remain superficial and public contributors do not—even if they choose to be involved in research—feel safe to share their experiences and opinions. An example of this is expressed by one of the refugees in the same study (Lampa et al. 2022): “Sometimes, perhaps, we say too much, will this upset you, that is what I am scared and worried about.” In addition, it might leave the public contributors feeling unheard and discouraged from further involvement—the opposite of empowerment. A final note is that researchers might not be aware of public contributors’ lack of trust, as this—ironically—requires some level of trust to express.

The root causes of forced migrants’ mistrust in government officials, are often rational and based on own experiences (Gandolfo 2022; Ni Raghallaigh 2014). Some forced migrants originate from contexts where the government do not act in the interest of the citizens and the safest action is to avoid contact. Some have difficult experiences from Swedish authorities, for example from the asylum process (van Eggermont Arwidson et al. 2022). Some have experienced racism and discrimination in other institutions, such as the healthcare system (Hamed et al. 2022). In addition, a fear of social services has been widespread among immigrants in Sweden in recent years, fuelled by occasions where the social services took immigrant children into compulsory care. This has led to lack of trust in government officials and how information is shared between government institutions.

If trust is not established—and especially public contributor’s trust in the researchers—the risk is that public contributors do not share their honest views. This jeopardizes the impact of PPI, as parts of the experience-based knowledge are not accessed and cannot be used in the project. Other important values in PPI, such as empowerment, is also at stake.

## Discussion

We have presented five themes of ethical issues in PPI with forced migrants. Involving forced migrants is in many ways similar to any involvement work—the values we aim for are the same and many of the issues we face are similar (Banks et al. 2013; Groot and Abma 2022). Likewise, many ethical issues are true for both previous studies and our findings, such as issues around communication (James 2023; Mitchell et al. 2019; Montreuil et al. 2019), trust and decision-making (Banks et al. 2013; Groot and Abma 2022; Pandya-Wood et al. 2017; Pincock and Bakunzi 2021), reimbursement (Montreuil et al. 2019; Pandya-Wood et al. 2017), and protection (Liabo et al. 2017). There are other issues in PPI, some of them of an ethical nature, that this form of data collection would not cover. Previously identified negative impacts of PPI that might be relevant ethical issues, such as increased costs (Oliver et al. 2019), burden of responsibility, feelings of overwork and frustration at limited opportunities to influence the research (Russell et al. 2020) were not identified in this article but are likely to have been present nonetheless.

We consider the identified ethical issues specific or exacerbated for PPI with forced migrants as they manifest themselves in a specific way with this population, in this context. One example is communication: this is challenging in most PPI collaboration, but with the added layer of initial mistrust and language interpretation, the risk of non-inclusive communication strategies leading to injustices is higher. Similarly, forced migrants are labelled as a vulnerable group in research ethics, which can contribute to researchers aiming to protect them more than they would a public contributor from the majority population—which in turn risks inhibiting their autonomy. Our findings highlight ethical issues that need to be acknowledged and actively counteracted to not disrupt the involvement processes with forced migrants.

In this article we analysed ethical issues in light of research ethics principles and PPI values. However, we found that these principles and values were not fully sufficient to discuss these ethical issues; we saw a need for an additional perspective. Here, we will discuss the identified issues using relational ethics. Relational ethics sees ethical actions as situated within relationships; ethics is something that happens between people and is central to relationships (Clandinin et al. 2018). Thereby, relational ethics “forces us to deal with real issues and social values” (Clandinin et al. 2018, 19). Banks et al. (2013) argue that the principle-based ethics and regulatory ethics processes is not enough for PPI projects; PPI also requires relational ethics i.e., approaches based on character and relationships, as a complement. A specific example of the usefulness of relational ethics is relational autonomy (Mackenzie and Stoljar 2000). The traditional principle of autonomy is focused on the individual and how they can or cannot take autonomous decisions (Mackenzie and Stoljar 2000). Relational autonomy, on the other hand, considers everyone’s social embeddedness and therefore looks at the social factors affecting autonomy. Mackenzie and Stoljar (2000) say that “identities are formed within the context of social relationships and shaped by a complex of intersecting social determinants, such as race, class, gender, and ethnicity.” Thus, the individual capacity for autonomy can only be realized within the context of relationships. From this point of view, both the ethical issues and the potential solutions, i.e., the opportunities for meaningful and ethical PPI, can be found within the relationships.

The idea of relational autonomy is highly relevant for our first ethical issue, “Treating forced migrant public contributors as a vulnerable group can inhibit autonomy.” This issue aligns with a longstanding discussion within the PPI community, which has been referred to as the “participation versus protection dilemma” (Liabo et al. 2017). In addition, this is a classic issue within medical ethics, often framed as paternalism-autonomy (Beauchamp and Childress 2013). In research ethics, protection has often been prioritized over participation and forced migrants might require an increased level of protection (Davidson, Hammarberg, and Fischer 2023). But within the PPI field, it is argued that being protected rather than involved can hinder important benefits coming out of involvement for both research, such as relevance, and public contributors, such as empowerment (Liabo et al. 2017). Ross et al. (2023) for example, found that peer researchers chose to become and stay involved in research, even though they had experiences of tokenistic approaches, where their input was not fully recognized, and expressions of systemic oppression, for example racism, within the academic system. The peer researchers were thereby fully aware that these risks existed in involvement but complied in order to make a difference. Therefore, underlying values of involvement, such as democratic representation or empowerment, also warrant consideration and might be more important to both researchers and public contributors. Applying relational autonomy to this ethical issue suggests that this is not necessarily a choice between participation or protection but rather that researchers need to provide the support needed for public contributors to autonomously make decisions for themselves. That said, this is not an easy task; it requires time and communication skills on the researchers’ behalf and a culture of shared decision-making (Gómez-Vírseda et al. 2019). This forms an example of when a relational ethical approach (Mackenzie and Stoljar 2000) is a useful approach; this is reliant on forming trusting relationships between the researchers and public contributors and taking responsibility for providing what public contributors need to be involved in a safe and meaningful way.

Avoiding tokenistic PPI is a key feature in PPI frameworks (Greenhalgh et al. 2019). Both using non-inclusive communication strategies and excluding public contributors from decision-making can be examples of tokenistic approaches. But even for participatory researchers with the best intentions, ethical and meaningful PPI can be challenging to deliver in practice (Egid et al. 2021; Pincock and Bakunzi 2021). PPI requires time, investment, and an interest from researchers, along with a genuine will to hand over a certain amount of power and control to the public contributors (Egid et al. 2021). In Sweden, as in other parts of the world, funders have started to request PPI in grant applications (Martineau et al. 2020). This means that researchers without experience of or support for PPI are required to include PPI in their work. Although this will likely increase involvement in research and could improve research quality, there is also a risk of an increase in tokenistic PPI which can negatively affect the conduct and sustainability of PPI initiatives. Ownership of and power over research is key here; this traditionally lies with the researcher but should—according to the logic of PPI—be shared with the public contributors. We find it important to acknowledge that tokenistic and unethically performed PPI can cause harm to public contributors (Martineau et al. 2020; Ross et al. 2023), and it is the researchers’ and institutions’ responsibility to prevent this. Ultimately, tokenistic PPI is likely not only harmful for research and for PPI as an approach but risks feeding into forced migrants’ experiences of participation in society. A common argument for PPI is that it can increase trust in the research community; similarly, tokenistic PPI where forced migrants are invited in but not fully involved, for example not being granted decision-making power, risks leading to the opposite—a decreased trust in the research community.

A key factor for PPI is institutional support, including allocation of time, resources, and practical support for PPI activities, as well as knowledge and academic support from colleagues at all levels within the organization. Lack of institutional support for PPI, as well as logistical and practical barriers, has been reported before (Martineau et al. 2020). For example, as public contributors can be reimbursed in many ways, local systems need to have guidelines for the ethical involvement of groups with increased vulnerability. Institutional norms and values can also affect PPI. Whether or not PPI values are established in the institutions where PPI is conducted, there are other norms and organizational factors at play within the same institutions. Previous studies have shown that health researchers experience expectations to work with PPI and want to do it well—while at the same time struggle with strict timelines, competitive research environments, and learning how to work with PPI (Thompson et al. 2009). Thereby, although they might assign to common PPI values (Gradinger et al. 2015) these internal values can clash with external norms. This can lead to moral distress (Jameton 1984) for participatory researchers, added to by the factor that PPI is often done by more junior researchers who are not necessarily in control of study processes (Groot and Abma 2022)*.* Institutional support is also essential to allow PPI researchers working in teams to include public contributors in decision-making; if decisions are to be taken together, everyone in the team must agree on this and be prepared to adjust accordingly.

Public contributors arrive to the collaboration with an own set of values and expectations. This article investigates ethical issues in light of PPI values, as identified by Gradinger et al (2015). These are values derived from the academic literature and not from forced migrants themselves, making it possible that these PPI values are not recognized by forced migrants. While this study did not specifically investigate values, the data suggests that relation-focused values, such as respect and trust, are valued by the involved forced migrants. Values are difficult to define and elicit, and it is likely to be more complicated to uncover the values that are driving the interaction when working with forced migrants where language barriers exist. Secondly, these values might not be the same as those of the researchers, which can cause value conflicts, and values will differ between involved groups. For these reasons, open discussions around values in the team, supported by value-eliciting tools and exercises, are needed to map values in each PPI context.

In the field of refugee studies, lack of trust for researchers and government officials among refugees has been reported and explored (Ni Raghallaigh 2014), which aligns with our findings. In the PPI context, Smith et al. (2022) report on the difficulties in and importance of trust-building processes in refugee youth involvement in sports research. Similarly, James (2023) reflects on the need to invest time in establishing trust in a participatory project with asylum seekers. There is a mistrust towards government officials, but this can be ameliorated through relationship-building beyond a strictly professional relationship; trust-building appears to be more important and more reliant on personal connection, in PPI with forced migrants compared to in PPI in general. Our findings indicated that involving forced migrants might need a prolonged process of trust-building and that it can be difficult for researchers to know which dimensions of trust have been established. This study did not specifically investigate the different dimensions of trust, but the data suggests that the trust that developed primarily consisted of what Freitag and Traunmüller (2009) calls “particularized trust” (i.e. an intimate trust in those in the immediate network) as the trusting relationships appeared when the public contributors had gotten to know the researchers personally. Freitag and Traunmüller (2009, 798) discuss how “generalized trust” and “particularized trust” interact, suggesting that “particularised trust is in fact a foundation upon which generalised trust can be built—given the right circumstances.” This provides some support to the often claimed increase in general trust in research as a result of PPI. Thus, investing in long-term partnerships is likely connected to more meaningful involvement of forced migrants.

The findings in this study build on years of experiences, discussions, observations, interviews, ethical reflections, and notetaking throughout projects utilizing PPI with forced migrants in health research. Our interest in exploring the specific factors of forced migrants’ involvement grew from our own experiences—we encountered situations we were not sure how to resolve and experienced moral distress (Jameton 1984) as participatory researchers. This is our attempt to concretize our empirical insights and view them through an ethical lens. The intention was to transform our experiences to highlight potential ethical issues and contribute to reflection for other teams working with PPI with forced migrants. The application of relational ethics in the discussion provided new perspectives on issues and potential solutions, for example to consider relational autonomy in addition to balancing participation and protection (Mackenzie and Stoljar 2000).

When initiating this article, we had already collected an abundance of data and reflected on ethical issues throughout the data collection. Using already collected data, and performing a secondary analysis, rather than collecting new data is ethically preferable to avoid contributing to research fatigue (Hinds et al. 2012). The vast amount and different kinds of data, collected by the authors, is a strength. Thematic analysis is a suitable method for data with large variations in richness (Braun and Clarke 2021). However, as with any secondary analysis, our findings are limited by the fact that the data were not collected to answer our specific research question; a data collection focused on ethical issues might have gained other insights.

We consider the empirical basis of this article a strength. Yet, it makes the findings context-specific and limits the transferability to other contexts. However, PPI will always be dependent on the context i.e., the political and organizational structures that either support the process or hinders it. One example is the immediate research environment where PPI is to be planned and conducted, which can be supportive around staff time, resources, knowledge, training etc. Secondly, the organizational level, e.g. the university, needs guidelines for reimbursement and for employing interpreters in a sustainable way. Yet another level is the funding bodies, which can set expectations for PPI funds and reimbursements. At both these levels, there are opportunities to support shared decision-making, by ensuring good knowledge of PPI at institutions and requiring thorough reporting of PPI initiatives when these are funded. The context for this article is a health research milieu at a Swedish university. PPI is still relatively new, but increasingly utilized, in the Nordic countries (Dengsø et al. 2023). This makes it important to investigate PPI in this context, as some issues might be context specific.

This article was co-written by academic researchers and public contributors who have a background in forced migration. One of these has experience of being a public contributor in health research and the other of working with research in a gatekeeper role. This is a strength of the article, as the contributions provided insight into ethical issues from both the researcher and public contributor perspective.

## Conclusion

In this article, we have presented five ethical issues in PPI with forced migrants, based on empirical data from PPI with forced migrants in health research. These are: Treating forced migrant public contributors as a vulnerable group can inhibit autonomy; Non-inclusive communication strategies can contribute to injustice; Regulations around payment risk excluding the most vulnerable from involvement; Public contributors risk being excluded from partaking in decision-making, and; If trust is not established, public contributors do not feel safe sharing honest input. While the findings align with the literature on PPI ethics, the identified ethical issues are exacerbated when involving forced migrants. Tokenistic and unethically conducted PPI can cause harm to public contributors and jeopardize PPI processes. The harms risk becoming worse in relation to forced migrants, a group already exposed to structural discrimination; it risks extending their experiences of mistrust and exploitation. Researchers and institutions need knowledge, planning, and structures to prevent these identified issues to lead to unethical PPI.

A relational ethics approach is useful for reflecting on ethical issues in PPI. The ethical issues are discussed as matters of relational ethics, where the issues arise within relationships. But relationships are also where the solutions are found, for example, regarding the development of trust which is closely connected to relationship-building within a PPI team. Ethical reflections are useful to understand how the underlying values and principles of both research and PPI contribute to the ethics in PPI with forced migrants. However, it is challenging for researchers to know how to work ethically with PPI, especially as many are now venturing into PPI collaborations based on funder requirements. We foresee a need for appropriate guidance for less-experienced researchers to develop the skills of carrying out PPI with a relational ethics approach. There is a need for more practically oriented, hands-on tools, based on relational ethics, for researchers and public contributors to use together, in order to build the trusting relationships and conduct PPI in ways that are beneficial for everyone in the team. Specifically, guidance on values elicitation and trust-building is needed. In addition, further research on trust in this context is needed, to explore how different dimensions of trust exist and interact in PPI. As PPI with forced migrants needs increased time for trust-building, funding and institutional support for this is needed.

## Data Availability

The participants in this study have not given consent for their data to be shared publicly. Due to the sensitive nature of the research supporting data is not available.
